# Supplementary figures and images for: Chronic exercise modulates the cellular immunity and its cannabinoid receptors expression
Source: PLoS One. 2019 Nov 18;14(11):e0220542. doi: 10.1371/journal.pone.0220542 (PMC6860935; doi:10.1371/journal.pone.0220542)

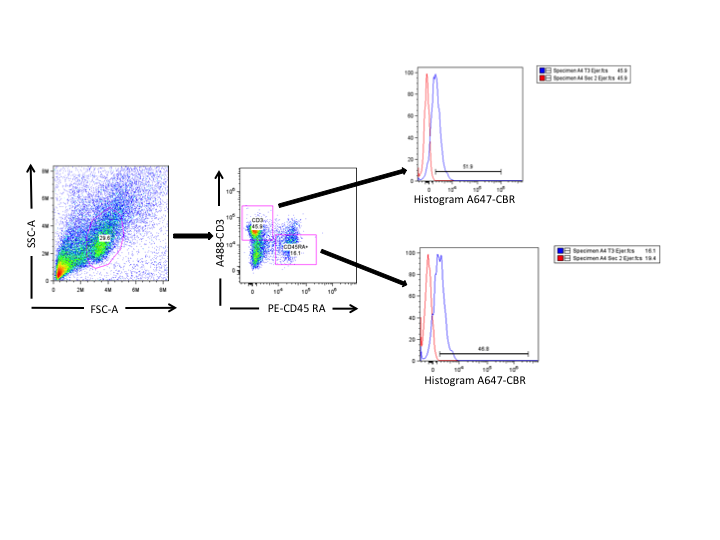

Supplement: S1 Fig — Single cell suspension was prepared and stained with fluorochrome-conjugated antibodies to separate splenocyte subpopulations and to mark cannabinoid receptors (CB1 and CB2). Data was analyzed with FlowJo software 8.7 for Mac. Lymphocytes were identified by their scatter properties (FSC-A x SSC-A plot). Splenocyte subpopulations were characterized by surface staining and gated for their quantity assessment. Subsequently each cellular subpopulation was analyzed for their expression of both cannabinoid receptors in their surface. (TIFF) [file pone.0220542.s001.tiff]

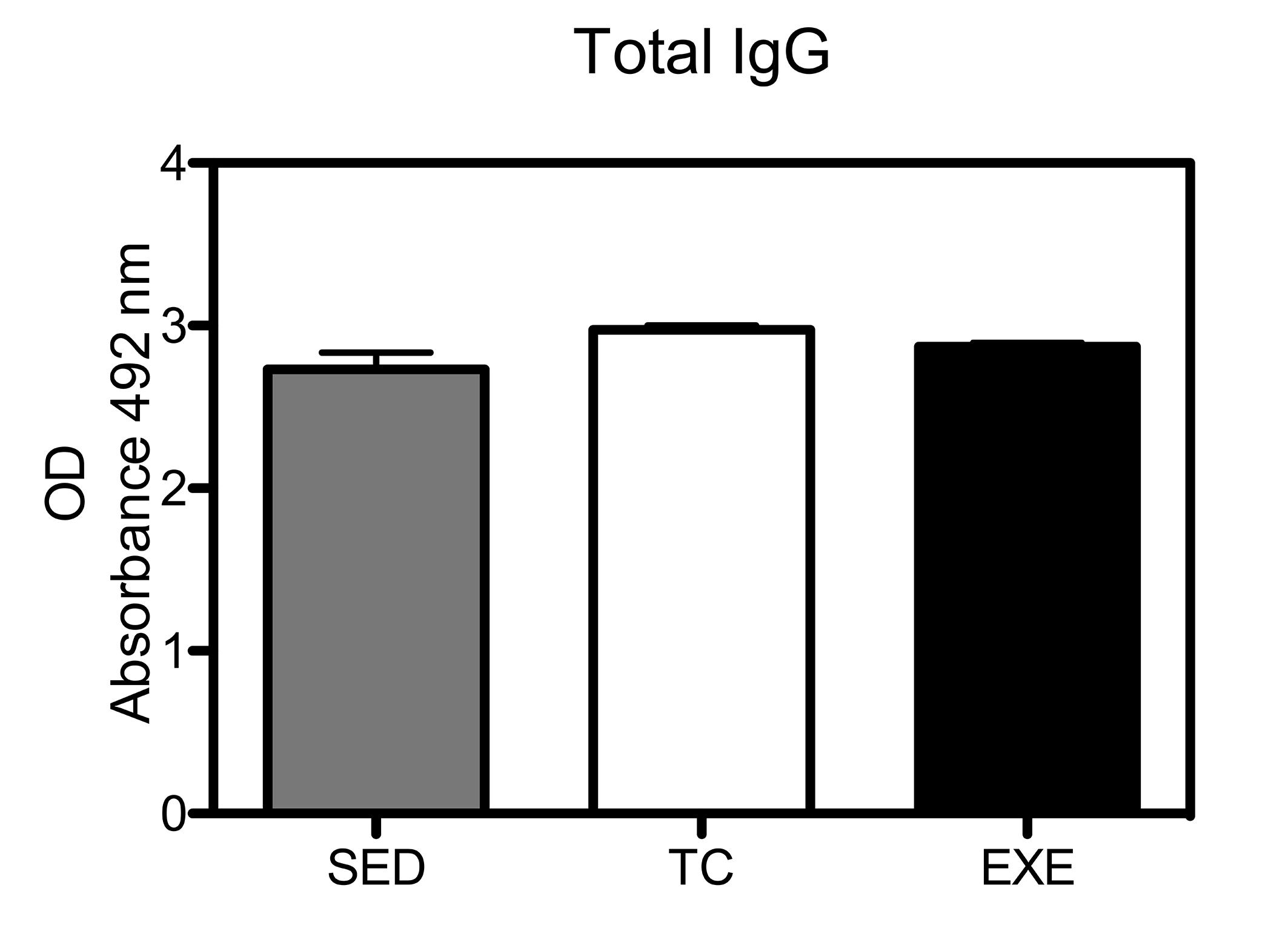

Supplement: S2 Fig — The analysis of total IgG was assessed for every experimental group with the use of a direct semi-quantitative ELISA. Statistical analysis did not show any significant difference among the experimental groups: SED (shaded bar), TC (white bar) and EXE (Solid bar). P>0.05. ANOVA, p = 0.0676, n = 6. (TIF) [file pone.0220542.s002.tif]

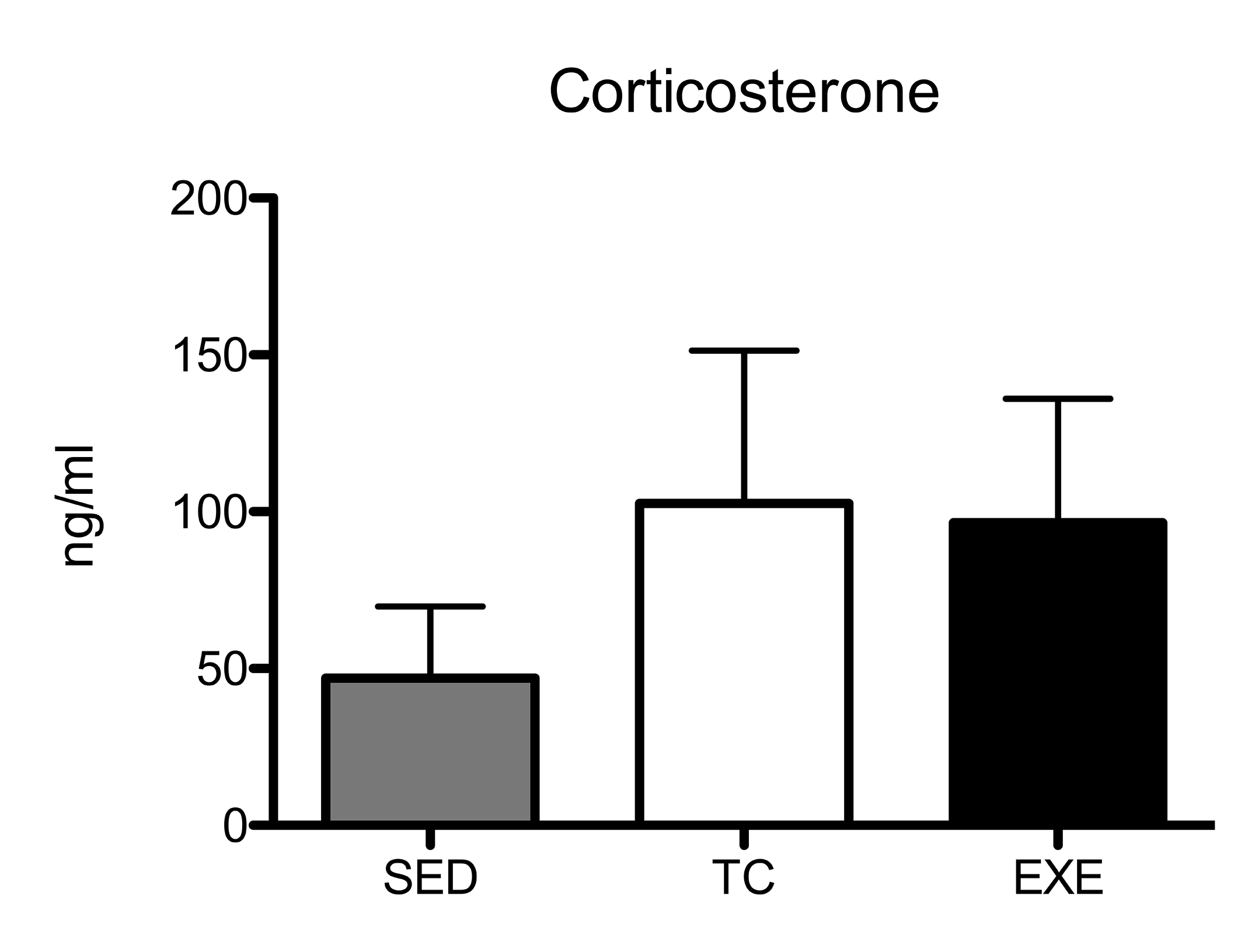

Supplement: S3 Fig — Data is shown as mean (ng/ml) +- SE for each group. There was no significant difference among group values in concentration of serum corticosterone. Groups analyzed: SED (shaded bar), TC (white bar) and EXE (Solid bar). When data was statistically analyzed (ANOVA, n = 6, p = 0.0473, Tukey’s) results show a significant difference among the experimental groups. Nonetheless, The post hoc analysis did not show any significant interaction among the experimental groups. Means of the groups: TC and EXE (102.6 and 96.5 ng/ml respectively) were notoriously higher than that of the SED group (46.8 ng/ml). (TIF) [file pone.0220542.s003.tif]
