# Supplementary material for: Chronic exercise modulates the cellular immunity and its cannabinoid receptors expression
Source: PLoS One. 2019 Nov 18;14(11):e0220542. doi: 10.1371/journal.pone.0220542 (PMC6860935; doi:10.1371/journal.pone.0220542)
Supplement: Attachment — Submitted filename: Manuscript ID PONE-D-19-20066-peer review.docx [file pone.0220542.s004.docx]

Manuscript ID PONE-D-19-20066

Cellular innate and adaptive immunity are affected by chronic exercise: implication of the cannabinergic system

by Salvador Valencia-Sánchez, Karen Nava-Castro, M. Isabel Palacios-Arreola, Oscar Prospéro-García, Jorge Morales-Montor, and René Drucker-Colín.

Comments to the Author:

The authors examined the effect of chronic treadmill exercise on cells of the immune system and cannabinoid receptors in male rats. These results suggested that chronic exercise can be recommended because it has beneficial effects on cellular innate and adaptive immunity. This paper is interesting to a wider audience across disciplines as immune systems and cannabinergic system. The authors suggest that the results contribute to the understanding of the benefits of exercise on the generally healthy person. Although these research approaches are interesting, to suggest the contribution to health there are several points that need to be clarified.

1. As basically data, it has need to show weight change in effect of Citrate Synthase activity (CS activity) of gastrocnemius muscle and/or weight changes about chronic treadmill exercise in rats. Because, in this experiment, it was conducted a chronic exercise at the vivo level, it needs information about training effect is necessary.

2. I would like to recommend that the authors need to observe the chronic exercise changes in cytokine production associated with the immunomodulation and inflammation about the functions of the other organs or tissues, immune cells.

3. For example, what about the effects of CRH by chronic training? In addition, it is necessary to add the data as to whether CRH changes by chronic training. Because the relationship between the changes in the immune system on exercise training is the effects of the relationship between the metabolic system, autonomic nervous system and endocrine system too. In particular, the exercise-induced cannabinoid is also involved in the central nerve, so it is thought that it is difficult to suggest health effects solely from the response of immunocyte and only receptor expression. Although I have no doubt about the quality of the presented work, I recommend revising the manuscript so that the purpose appears more clearly.

4. I would like to recommend that the authors demonstrate to investigate the immunocyte functional effect of chronic exercise by cannabinoid receptor antagonist, virodhamine administration.

Minor comments:

1. P4,L4: “physycal activity” = “physical activity”
