# Supplementary material for: Chronic exercise modulates the cellular immunity and its cannabinoid receptors expression
Source: PLoS One. 2019 Nov 18;14(11):e0220542. doi: 10.1371/journal.pone.0220542 (PMC6860935; doi:10.1371/journal.pone.0220542)
Supplement: Attachment — Submitted filename: Rebbutal Letter .docx [file pone.0220542.s006.docx]

**Rebuttal letter**

**Reviewer #2:**

1- The authors have summarized the title, but the main tissue (endocannabinoid system) was excluded.

Reply: Thank you for the observation, the title has now been changed to “Chronic exercise modulates the cellular immunity and its cannabinoid receptors expression”, including the cannabinoid system in the title once again.

2- Ok!

3- At the end of the introduction the authors describe hypothesis, as suggested, but now the study objectives are omitted.

Reply: We had already included the objectives of our work before the hypotesis. nontheless, in order to make it clearer we have changed it to:

“Altogether, The objective of our study is to explore the long-term changes that chronic exercise (CE) produces in the proportion of splenocytes from the adaptive and innate immunity, and to assess the effects that it has on their function (by performing proliferation tests and cytotoxicity test with total splenocytes *in vitro*), and finally to determine if the expression of CBR in these cells is affected by this model of exercise.”

4- When asked the authors whether exercise activates or inhibits the immune system for the purpose of describing in the introduction, they replied that there is no study, but I suggest checking the review and describing any study addressed by it (10.3389/fimmu.2018.00648).

Reply: we added recent information envolving the interaction between exercise, metabolic deseases and cancer, this published data even suggest its use as an alternative therapy. This addition can be found at the end of the first paraghaph of the introduction as previously requested.

About the interesting review suggested, some lines have been added on the introduction making reference to this work and its take homme message about a new interpretation of older data, and the implications it has on the understanding of the beneficial physiological changes that the organisms undoergoes during the physical activity.

5- About weight of the animals, its ok!

6- About electrical stimulus, its ok!

7- The table 1 figure legend need to describe each abbreviation. Ok about requested table 1.

Reply: the abbreviations have been added.

8- The last question about cb2 receivers should be included in the discussion. It is important to note that the results of the literature on western blot receptor-enhanced cb2 receptors and immunofluorescence are relevant and cannot be lowered compared to cytometry, even though cytometry has good accuracy.

Reply: According to the question previously made, we have added a couple of lines in the cannabinoid receptors section (in the dicussion), where we explain the need for a double and independent staining for every cell suspention aliquot in order to assess the exprssion of each CBR due to the shared epitope for the secondary antibody.

We have also added some references to previous data regarding the assesment of CBr through different techniques, like western blot and ummunohistochemestry, highlighting our position with respect to the validity of such techniques and the information they provide.

**Reviewer #3:** The main objective of this study was to evaluate the effect of chronic treadmill exercise on lymphocytes, NK cells and macrophages of male rats investigating possible alterations of cannabinoid receptors in this process. The study is very interesting and important to better understanding of chronic exercise in immune cells. However, there are some minor points that need to be addressed.

1. The title need to be changed to indicate the important effects on cannabinoids receptors.

Reply: Thanks for the observation, we have includded the cannabinoid receptors on the title.

2. The purpose of the study and the main hypothesis is not clear in the abstract.

Reply: the objectves and hypohesis have been added as requested.

3. The identification of CD11b is sufficiently to identify macrophages?

Reply: This is an important observation.

The expression of CD11b is not exclusive of macrophages, it is shared among different cells with myeloid origin, but once we restrict their identification to big and coplex cells, we obtain a reasonably adecuate identification of this cell subset.

This aclaration has been added in the section of “Materials and methods” and the subsection “Flow Cytometry”.

4. The figures quality need to be improved.

Reply: the images lost quality when converted to .tiff the first time, making some lines disapear or blurry. That has been fixed. Now they have been converted to eps. format which alowed us to improve the resolution to 500 dpis, whitout exceeding the 10mb limit for the upload.
